# Supplementary material for: HIVprotI: an integrated web based platform for prediction and design of HIV proteins inhibitors
Source: J Cheminform. 2018 Mar 9;10:12. doi: 10.1186/s13321-018-0266-y (PMC5845081; doi:10.1186/s13321-018-0266-y)
Supplement: Supplementary file 2 — Additional file 2. Source code of HIVProtI web server. [file 13321_2018_266_MOESM2_ESM.zip › HIVprotI_Source-code/hivprotI/weka-3-6-6/changelogs/CHANGELOG-3-5-3.html]

Weka-3-5-3 Changelog


# Weka-3-5-3 Changelog

Designed for use with Ant.

---

2006-06-08 02:22 eibe

```
updated
```

- core/version.txt (1.2)

2006-06-07 22:38 fracpete

```
getDelimiters/setDelimiters now always escape/unescape the double and single quote - fixes problem with saving an Experimenter result via JDBC
```

- filters/unsupervised/attribute/StringToWordVector.html (1.13)

2006-06-07 22:36 fracpete

```
fixed Javadoc
splitOptions no longer throws an exception for escaped chars other than " and \, eg \n and \t
joinOptions uses now backQuoteChars - option can then be used again in splitOptions and/or on commandline/SimpleCLI
```

- core/Utils.html (1.53)

2006-06-07 22:32 fracpete

```
keys are now always surrounded by double quotes instead of single quotes and content is escaped with Utils.backQuoteChars(String) - fixes problem with StringToWordVector filter
```

- experiment/DatabaseResultListener.html (1.10)
- experiment/DatabaseUtils.html (1.24)

2006-06-07 22:22 fracpete

```
added menu item to enter the configuration manually (besides copying it to the clipboard and showing the properties)
```

- gui/PropertyPanel.html (1.11)

2006-06-07 22:20 fracpete

```
added serialVersionUID
cleaned up imports
removed custom splitOptions method with the one from the Utils class
```

- gui/SimpleCLI.html (1.8)

2006-06-07 03:37 fracpete

```
rewritten to make it useable in the AbstractClassifierTest JUnit test class
```

- core/CheckOptionHandler.html (1.9)

2006-06-01 20:35 fracpete

```
fixed calculation of correlation coefficient: for very small numbers, Utils.smOrEq() returned 0 which resulted in a correlation coefficient of 0 instead of e.g. 0.89
```

- classifiers/Evaluation.html (1.62)

2006-06-01 06:36 fracpete

```
replaced tab, cr and lf with entities - avoids problems in options of OptionHandlers
```

- core/xml/XMLSerialization.html (1.13)

2006-05-30 04:04 fracpete

```
input and output formats are only set if it's still the first batch of instances (i.e., m_FirstBatchDone == false)
```

- filters/MultiFilter.html (1.4)
- filters/SimpleBatchFilter.html (1.3)
- filters/SimpleStreamFilter.html (1.4)

2006-05-30 03:02 fracpete

```
revoked modifications regarding copying of attributes in Instances(Instances,int) constructor (resulted in higher memory consumption) - AttributeLocators do the trick now
```

- core/Instances.html (1.65)

2006-05-30 01:58 fracpete

```
added missing attribute types for attribute info in parentheses, e.g, (Rel)
```

- gui/boundaryvisualizer/BoundaryVisualizer.html (1.15)

2006-05-30 01:54 fracpete

```
added missing attribute types for attribute info in parentheses, e.g, (Rel)
```

- gui/visualize/MatrixPanel.html (1.13)

2006-05-30 01:54 fracpete

```
added missing attribute types for attribute info in parentheses, e.g, (Rel)
```

- gui/visualize/VisualizePanel.html (1.26)

2006-05-30 01:53 fracpete

```
added missing attribute types for attribute info in parentheses, e.g, (Rel)
```

- gui/AttributeSummaryPanel.html (1.10)

2006-05-30 01:53 fracpete

```
added missing attribute types for attribute info in parentheses, e.g, (Rel)
```

- gui/AttributeVisualizationPanel.html (1.24)

2006-05-30 01:52 fracpete

```
added missing attribute types for attribute info in parentheses, e.g, (Rel)
```

- gui/explorer/ClassifierPanel.html (1.90)

2006-05-30 01:52 fracpete

```
added missing attribute types for attribute info in parentheses, e.g, (Rel)
```

- gui/beans/ClassAssignerCustomizer.html (1.4)

2006-05-30 01:52 fracpete

```
added missing attribute types for attribute info in parentheses, e.g, (Rel)
```

- gui/explorer/ClustererPanel.html (1.50)

2006-05-30 01:51 fracpete

```
added missing attribute types for attribute info in parentheses, e.g, (Rel)
```

- gui/explorer/AssociationsPanel.html (1.21)

2006-05-30 01:51 fracpete

```
added missing attribute types for attribute info in parentheses, e.g, (Rel)
```

- gui/explorer/AttributeSelectionPanel.html (1.38)

2006-05-30 01:26 fracpete

```
replaced weka.classifiers.RandomizableClassifier with weka.core.Randomizable
```

- classifiers/meta/AdaBoostM1.html (1.32)

2006-05-30 01:19 fracpete

```
added m_MaxDepth parameter to control the maximum depth of the tree (additional parameter in buildTree for the current level: depth)
adapted setOptions/getOptions/listOptions
getOptions method uses now a Vector instead of an array to assemble the options
```

- classifiers/trees/RandomForest.html (1.10)

2006-05-30 01:17 fracpete

```
added m_MaxDepth parameter to control the maximum depth of the tree (additional parameter in buildTree for the current level: depth)
removed m_Debug since it overrides the variable from the Classifier class and adapted setOptions/getOptions/listOptions
getOptions method uses now a Vector instead of an array to assemble the options
```

- classifiers/trees/RandomTree.html (1.14)

2006-05-24 05:48 fracpete

```
fixed Javadoc
```

- experiment/PairedCorrectedTTester.html (1.10)

2006-05-24 01:31 fracpete

```
fixed Technical Information
updated Javadoc
```

- classifiers/trees/LMT.html (1.8)

2006-05-24 01:28 fracpete

```
fixed Technical Information
updated Javadoc
```

- classifiers/functions/SimpleLogistic.html (1.10)

2006-05-24 00:49 fracpete

```
fixed Javadoc ("incproceedings" typo)
```

- datagenerators/clusterers/BIRCHCluster.html (1.5)

2006-05-24 00:48 fracpete

```
error message of unknown is now output on System.err - otherwise problems with Javadoc generation via AllJavadoc
```

- core/stemmers/SnowballStemmer.html (1.6)

2006-05-24 00:48 fracpete

```
fixed Javadoc (display of technical information of a journal)
```

- core/stemmers/IteratedLovinsStemmer.html (1.5)
- core/stemmers/LovinsStemmer.html (1.5)

2006-05-24 00:43 fracpete

```
fixed Javadoc
```

- core/converters/DatabaseLoader.html (1.6)
- core/converters/DatabaseSaver.html (1.5)

2006-05-24 00:42 fracpete

```
fixed Javadoc ("incproceedings" typo)
```

- clusterers/DBScan.html (1.5)
- clusterers/OPTICS.html (1.5)
- clusterers/XMeans.html (1.14)

2006-05-24 00:41 fracpete

```
fixed Javadoc ("incproceedings" typo)
```

- classifiers/trees/ADTree.html (1.5)
- classifiers/trees/M5P.html (1.6)
- classifiers/trees/NBTree.html (1.7)

2006-05-24 00:39 fracpete

```
fixed Javadoc ("incproceedings" typo)
```

- classifiers/rules/DecisionTable.html (1.34)
- classifiers/rules/M5Rules.html (1.8)

2006-05-24 00:39 fracpete

```
fixed Javadoc
```

- classifiers/rules/JRip.html (1.17)

2006-05-24 00:38 fracpete

```
fixed Javadoc ("incproceedings" typo)
```

- classifiers/misc/FLR.html (1.7)
- classifiers/misc/VFI.html (1.12)

2006-05-24 00:37 fracpete

```
fixed Javadoc ("incproceedings" typo)
```

- classifiers/mi/CitationKNN.html (1.3)
- classifiers/mi/MIBoost.html (1.3)
- classifiers/mi/MIOptimalBall.html (1.3)

2006-05-24 00:36 fracpete

```
fixed Javadoc
```

- classifiers/mi/MIEMDD.html (1.4)

2006-05-24 00:35 fracpete

```
fixed Javadoc ("incproceedings" typo)
```

- classifiers/meta/Decorate.html (1.7)
- classifiers/meta/END.html (1.5)
- classifiers/meta/Grading.html (1.10)
- classifiers/meta/MetaCost.html (1.21)
- classifiers/meta/OrdinalClassClassifier.html (1.15)
- classifiers/meta/StackingC.html (1.11)

2006-05-24 00:34 fracpete

```
fixed Javadoc (display of technical information of a journal)
```

- classifiers/meta/MultiBoostAB.html (1.11)

2006-05-24 00:33 fracpete

```
fixed Javadoc
```

- classifiers/meta/Dagging.html (1.4)

2006-05-24 00:29 fracpete

```
fixed Javadoc ("incproceedings" typo)
```

- classifiers/meta/nestedDichotomies/ClassBalancedND.html (1.5)
- classifiers/meta/nestedDichotomies/DataNearBalancedND.html (1.5)
- classifiers/meta/nestedDichotomies/ND.html (1.5)

2006-05-24 00:28 fracpete

```
fixed Javadoc ("incproceedings" typo)
```

- classifiers/lazy/KStar.html (1.6)
- classifiers/lazy/LWL.html (1.16)

2006-05-24 00:26 fracpete

```
fixed Javadoc ("incproceedings" typo)
```

- classifiers/functions/PaceRegression.html (1.6)
- classifiers/functions/VotedPerceptron.html (1.20)

2006-05-24 00:23 fracpete

```
fixed Javadoc
```

- classifiers/functions/IsotonicRegression.html (1.2)

2006-05-24 00:23 fracpete

```
fixed Javadoc (removed umlaut from technical information)
```

- classifiers/functions/supportVector/StringKernel.html (1.2)

2006-05-24 00:22 fracpete

```
fixed Javadoc ("incproceedings" typo)
```

- classifiers/bayes/ComplementNaiveBayes.html (1.5)
- classifiers/bayes/HNB.html (1.7)
- classifiers/bayes/NaiveBayes.html (1.19)
- classifiers/bayes/NaiveBayesMultinomial.html (1.12)
- classifiers/bayes/NaiveBayesUpdateable.html (1.7)

2006-05-24 00:20 fracpete

```
fixed Javadoc
```

- classifiers/bayes/net/search/local/LocalScoreSearchAlgorithm.html (1.4)

2006-05-24 00:20 fracpete

```
fixed Javadoc (display of technical information of a journal)
```

- classifiers/bayes/net/search/local/TAN.html (1.5)

2006-05-24 00:19 fracpete

```
fixed Javadoc (display of technical information of a journal)
```

- classifiers/bayes/net/search/global/TAN.html (1.5)

2006-05-24 00:18 fracpete

```
fixed Javadoc ("incproceedings" typo)
```

- classifiers/BVDecompose.html (1.12)
- classifiers/BVDecomposeSegCVSub.html (1.5)

2006-05-24 00:17 fracpete

```
fixed Javadoc ("incproceedings" typo)
```

- attributeSelection/ConsistencySubsetEval.html (1.12)
- attributeSelection/FCBFSearch.html (1.4)
- attributeSelection/RaceSearch.html (1.18)
- attributeSelection/RandomSearch.html (1.15)
- attributeSelection/ReliefFAttributeEval.html (1.18)
- attributeSelection/SymmetricalUncertAttributeSetEval.html (1.4)

2006-05-24 00:11 fracpete

```
fixed Javadoc ("incproceedings" typo)
```

- associations/Apriori.html (1.25)
- associations/PredictiveApriori.html (1.7)

2006-05-23 21:59 fracpete

```
brought up-to-date
```

- gui/GenericObjectEditor.props (1.113)

2006-05-23 21:23 fracpete

```
added excludes for the bayes net package (from Remco)
```

- gui/GenericPropertiesCreator.excludes (1.2)

2006-05-23 05:33 fracpete

```
applied Eibe's fix regarding missing values and class attribute
```

- filters/unsupervised/attribute/AbstractTimeSeries.html (1.5)
- filters/unsupervised/attribute/TimeSeriesDelta.html (1.4)
- filters/unsupervised/attribute/TimeSeriesTranslate.html (1.5)

2006-05-22 03:24 fracpete

```
if class attribute is set, it is skipped now
fixed loss of class attribute (problems with FilteredClassifier otherwise)
```

- filters/unsupervised/attribute/FirstOrder.html (1.6)

2006-05-22 02:23 fracpete

```
removed obsolete TODO
```

- TODO.dtd (1.2)
- TODO.xml (1.2)

2006-05-22 01:58 fracpete

```
added "*.excludes" to targets "compile" and "srcjar"
added "*.xml" to target "srcjar" to also add the build.xml file to the jar
moved CVSROOT to variable "cvsroot_anon" (easier maintenance)
```

- build.xml (1.29)

2006-05-22 00:53 fracpete

```
cleaned up imports
added serialVersionUIDs
JList now of type SINGLE_SELECTION to avoid confusion
```

- gui/ResultHistoryPanel.html (1.22)

2006-05-19 04:05 fracpete

```
replaced the hack for the ResultListeners to suppress the listing of ResultProducers with a general framework for suppressing Interfaces, Superclasses or explicit Classes
fixed Javadoc
prefixed members with "m_"
```

- gui/GenericPropertiesCreator.html (1.9)

2006-05-18 06:45 fracpete

```
fixed Javadoc
```

- core/Memory.html (1.4)

2006-05-18 05:50 fracpete

```
added a "makeCopies" method for convenience
```

- filters/Filter.html (1.29)

2006-05-15 04:05 fracpete

```
words and word-separators for string generation are now options, which enables one to generate strings with a certain alphabet (e.g., for gene sequences) and structure (e.g., no whitespaces/separators) - otherwise checks for biological algorithms failed due to malformed input strings
```

- classifiers/CheckClassifier.html (1.24)

2006-05-15 04:01 fracpete

```
words and word-separators for string generation are now options, which enables one to generate strings with a certain alphabet (e.g., for gene sequences) and structure (e.g., no whitespaces/separators)
```

- core/TestInstances.html (1.3)

2006-05-14 21:18 fracpete

```
added "Resize" button (suggested by Andriy Palamarchuk)
added serialVersionUIDs
cleaned up imports
fixed Javadoc
```

- gui/CostMatrixEditor.html (1.8)

2006-05-12 04:24 fracpete

```
added additional forInstances method, to detect multi-instance data automatically based on the structure of the attributes (3 attributes: nominal=bag-id,relational=bag,other=class)
```

- core/Capabilities.html (1.7)

2006-05-12 03:24 fracpete

```
added "-g <file>" option for outputting the graph representation of Drawable clusterers
```

- clusterers/ClusterEvaluation.html (1.30)

2006-05-09 23:13 fracpete

```
added links to PS/PDF files
```

- classifiers/functions/SMO.html (1.61)

2006-05-07 23:08 fracpete

```
fixed toString() method - thanks to JosÈ Marco de la Rosa
```

- core/matrix/Matrix.html (1.5)

2006-05-04 22:35 fracpete

```
uses now the new copyValues methods instead of the old copyStringValues ones
```

- filters/supervised/attribute/Discretize.html (1.5)
- filters/supervised/attribute/NominalToBinary.html (1.5)
- filters/unsupervised/attribute/Add.html (1.4)
- filters/unsupervised/attribute/AddCluster.html (1.5)
- filters/unsupervised/attribute/AddExpression.html (1.4)
- filters/unsupervised/attribute/AddID.html (1.3)

2006-05-04 22:35 fracpete

```
uses now the new copyValues methods instead of the old copyStringValues ones
```

- filters/unsupervised/attribute/Copy.html (1.4)
- filters/unsupervised/attribute/Discretize.html (1.9)
- filters/unsupervised/attribute/MultiInstanceToPropositional.html (1.3)
- filters/unsupervised/attribute/NominalToBinary.html (1.9)
- filters/unsupervised/attribute/PropositionalToMultiInstance.html (1.3)
- filters/unsupervised/attribute/Remove.html (1.6)
- filters/unsupervised/attribute/Reorder.html (1.3)

2006-05-04 22:24 fracpete

```
extended to be more flexible regarding attribute ranges - necessary for filters that add/remove/change attributes
```

- core/AttributeLocator.html (1.2)
- core/RelationalLocator.html (1.1)
- core/StringLocator.html (1.2)

2006-05-04 22:20 fracpete

```
Hashtable is now also used for relational values (otherwise values will be added even though already existing)
closing name of relational attribute in header gets now quoted if necessary
```

- core/Attribute.html (1.40)

2006-05-03 21:24 fracpete

```
added technical reference
```

- classifiers/rules/Ridor.html (1.15)

2006-05-02 03:08 fracpete

```
correct classIndex is set before ArffViewer panel is displayed
```

- gui/explorer/PreprocessPanel.html (1.58)

2006-05-02 00:43 fracpete

```
fixed display of attribute name for relational popup-dialog
```

- gui/arffviewer/ArffTable.html (1.6)

2006-05-01 05:22 fracpete

```
fixed getOptions for stepsize and startpoint (leading - of option was missing)
```

- attributeSelection/RankSearch.html (1.14)

2006-04-30 22:09 fracpete

```
added tiptext for maximumAttributeNames
fixed Javadoc
```

- attributeSelection/PrincipalComponents.html (1.31)

2006-04-27 06:43 fracpete

```
fixed Javadoc
```

- experiment/ResultMatrix.html (1.7)

2006-04-27 06:42 fracpete

```
fixed technical information
update Javadoc
```

- datagenerators/classifiers/classification/Agrawal.html (1.4)

2006-04-27 06:41 fracpete

```
fixed Javadoc
```

- gui/visualize/JComponentWriter.html (1.2)
- gui/visualize/JPEGWriter.html (1.2)
- gui/visualize/PostscriptGraphics.html (1.3)

2006-04-27 06:41 fracpete

```
fixed Javadoc
```

- core/xml/XMLDocument.html (1.4)

2006-04-27 06:21 fracpete

```
fixed Javadoc
```

- gui/ComponentHelper.html (1.2)

2006-04-27 06:21 fracpete

```
fixed Javadoc
```

- core/xml/KOML.html (1.3)
- core/xml/XMLSerializationMethodHandler.html (1.4)

2006-04-27 06:19 fracpete

```
fixed Javadoc
```

- filters/unsupervised/instance/RemoveWithValues.html (1.10)

2006-04-27 06:05 fracpete

```
fixed Javadoc
```

- core/xml/XMLBasicSerialization.html (1.5)

2006-04-27 06:05 fracpete

```
fixed Javadoc
```

- experiment/xml/XMLExperiment.html (1.5)

2006-04-27 06:04 fracpete

```
fixed Javadoc
```

- filters/unsupervised/attribute/MergeTwoValues.html (1.6)
- filters/unsupervised/attribute/StringToNominal.html (1.6)

2006-04-27 05:56 fracpete

```
updated and fixed Javadoc
```

- filters/unsupervised/attribute/SwapValues.html (1.6)

2006-04-27 05:55 fracpete

```
updated Javadoc
```

- filters/supervised/instance/StratifiedRemoveFolds.html (1.3)

2006-04-27 05:27 fracpete

```
added serialVersionUID
fixed Javadoc
```

- classifiers/functions/pace/PaceMatrix.html (1.3)

2006-04-27 05:09 fracpete

```
fixed Javadoc
added technical information
```

- classifiers/functions/pace/ChisqMixture.html (1.3)
- classifiers/functions/pace/MixtureDistribution.html (1.3)
- classifiers/functions/pace/NormalMixture.html (1.3)

2006-04-27 04:31 fracpete

```
fixed Javadoc
```

- classifiers/functions/LeastMedSq.html (1.13)

2006-04-27 04:29 fracpete

```
cleaned up imports
fixed Javadoc
```

- classifiers/evaluation/ThresholdCurve.html (1.20)

2006-04-27 04:23 fracpete

```
cleaned up imports
fixed Javadoc
```

- classifiers/evaluation/CostCurve.html (1.7)

2006-04-27 04:19 fracpete

```
cleaned up imports
added serialVersionUID
fixed Javadoc
```

- classifiers/bayes/net/ParentSet.html (1.5)

2006-04-27 04:18 fracpete

```
added technical information
```

- classifiers/bayes/net/ADNode.html (1.5)

2006-04-27 04:00 fracpete

```
cleaned up imports
added serialVersionUID
fixed Javadoc
removed obsolete code
```

- attributeSelection/AttributeSelection.html (1.40)

2006-04-27 03:51 fracpete

```
cleaned up imports
added serialVersionUID
fixed Javadoc
```

- associations/AprioriItemSet.html (1.4)

2006-04-27 03:31 fracpete

```
cleaned up imports
added serialVersionUID
added tags for Javadoc automation
```

- filters/unsupervised/attribute/AddNoise.html (1.4)
- filters/unsupervised/attribute/ChangeDateFormat.html (1.4)
- filters/unsupervised/attribute/ClusterMembership.html (1.8)
- filters/unsupervised/attribute/MakeIndicator.html (1.3)
- filters/unsupervised/attribute/MathExpression.html (1.4)
- filters/unsupervised/attribute/Normalize.html (1.5)
- filters/unsupervised/attribute/NumericToBinary.html (1.4)
- filters/unsupervised/attribute/NumericTransform.html (1.6)
- filters/unsupervised/attribute/Obfuscate.html (1.3)
- filters/unsupervised/attribute/PotentialClassIgnorer.html (1.3)
- filters/unsupervised/attribute/RemoveType.html (1.5)
- filters/unsupervised/attribute/RemoveUseless.html (1.6)
- filters/unsupervised/attribute/ReplaceMissingValues.html (1.5)
- filters/unsupervised/attribute/Standardize.html (1.5)

2006-04-27 03:31 fracpete

```
cleaned up imports
added serialVersionUID
added tags for Javadoc automation
```

- filters/unsupervised/instance/NonSparseToSparse.html (1.2)
- filters/unsupervised/instance/Normalize.html (1.4)
- filters/unsupervised/instance/Randomize.html (1.3)
- filters/unsupervised/instance/RemoveFolds.html (1.2)
- filters/unsupervised/instance/RemoveFrequentValues.html (1.2)
- filters/unsupervised/instance/RemoveMisclassified.html (1.4)
- filters/unsupervised/instance/RemovePercentage.html (1.4)
- filters/unsupervised/instance/RemoveRange.html (1.4)
- filters/unsupervised/instance/Resample.html (1.5)
- filters/unsupervised/instance/SparseToNonSparse.html (1.3)

2006-04-27 03:30 fracpete

```
cleaned up imports
added serialVersionUID
added tags for Javadoc automation
```

- filters/supervised/attribute/AttributeSelection.html (1.4)
- filters/supervised/attribute/ClassOrder.html (1.5)

2006-04-27 03:30 fracpete

```
cleaned up imports
added serialVersionUID
added tags for Javadoc automation
```

- filters/supervised/instance/Resample.html (1.5)
- filters/supervised/instance/SpreadSubsample.html (1.4)

2006-04-27 03:29 fracpete

```
cleaned up imports
added serialVersionUID
added tags for Javadoc automation
added technical information
```

- filters/unsupervised/attribute/PKIDiscretize.html (1.5)

2006-04-27 03:29 fracpete

```
cleaned up imports
added serialVersionUID
added tags for Javadoc automation
added technical information
```

- filters/unsupervised/attribute/RandomProjection.html (1.5)

2006-04-26 06:17 fracpete

```
fixed "imperfect" output if pages are missing
```

- core/TechnicalInformation.html (1.6)

2006-04-12 21:12 mhall

```
Fixed bug in rule output. Second number in the brackets at a leaf now reports the rms error as a percentage of the global standard deviation.
```

- classifiers/trees/m5/Rule.html (1.11)

2006-04-12 21:02 fracpete

```
svm_parameter.degree is no longer a double but an int (version 2.82)
added technical information about LIBSVM
updated Javadoc
```

- classifiers/functions/LibSVM.html (1.5)

2006-04-10 23:31 fracpete

```
fixed technical information (removed pp/vol/no for articles)
```

- clusterers/Cobweb.html (1.20)
- clusterers/FarthestFirst.html (1.5)

2006-04-10 23:30 fracpete

```
fixed technical information (removed pp/vol/no for articles)
```

- classifiers/trees/Id3.html (1.18)

2006-04-10 23:30 fracpete

```
fixed technical information (URL -> PS/PDF)
```

- classifiers/trees/UserClassifier.html (1.24)

2006-04-10 23:29 fracpete

```
fixed technical information (removed pp/vol/no for articles)
```

- classifiers/rules/OneR.html (1.20)
- classifiers/rules/Prism.html (1.20)

2006-04-10 23:29 fracpete

```
fixed technical information (URL -> PS/PDF)
```

- classifiers/rules/PART.html (1.6)

2006-04-10 23:28 fracpete

```
fixed technical information (removed pp/vol/no for articles)
```

- classifiers/meta/Bagging.html (1.36)
- classifiers/meta/ClassificationViaRegression.html (1.24)
- classifiers/meta/Stacking.html (1.28)

2006-04-10 23:27 fracpete

```
fixed technical information (URL -> PS/PDF)
```

- classifiers/meta/LogitBoost.html (1.36)

2006-04-10 23:26 fracpete

```
fixed technical information (URL -> PS/PDF)
```

- classifiers/meta/AdditiveRegression.html (1.20)

2006-04-10 23:26 fracpete

```
fixed technical information (removed pp/vol/no for articles)
```

- classifiers/lazy/IB1.html (1.17)
- classifiers/lazy/IBk.html (1.37)
- classifiers/lazy/LBR.html (1.9)

2006-04-10 23:24 fracpete

```
fixed technical information (removed pp/vol/no for articles)
```

- classifiers/functions/Logistic.html (1.35)

2006-04-10 23:24 fracpete

```
fixed technical information (removed pp/vol/no for articles)
```

- classifiers/functions/Winnow.html (1.11)

2006-04-10 23:23 fracpete

```
fixed technical information (removed pp/vol/no for articles)
```

- classifiers/bayes/net/search/local/K2.html (1.5)

2006-04-10 23:23 fracpete

```
fixed technical information (removed pp/vol/no for articles)
```

- classifiers/bayes/net/search/global/K2.html (1.5)

2006-04-10 23:22 fracpete

```
fixed technical information (removed pp/vol/no for articles)
```

- attributeSelection/SVMAttributeEval.html (1.21)
- attributeSelection/WrapperSubsetEval.html (1.26)

2006-04-10 23:22 fracpete

```
fixed technical information (removed pp/vol/no for articles)
```

- classifiers/bayes/AODE.html (1.15)

2006-04-10 23:20 fracpete

```
fixed technical information (removed pp/vol/no for articles)
```

- associations/Tertius.html (1.6)

2006-04-10 23:18 fracpete

```
Kernels are now accessible via GenericObjectEditor, as well as a parameter from commandline
```

- classifiers/mi/MISMO.html (1.3)
- classifiers/mi/MISVM.html (1.3)

2006-04-10 23:17 fracpete

```
Kernels are now accessible via GenericObjectEditor, as well as a parameter from commandline
```

- classifiers/functions/GaussianProcesses.html (1.4)
- classifiers/functions/SMOreg.html (1.11)

2006-04-10 23:17 fracpete

```
Kernels are now accessible via the GenericObjectEditor and OptionHandlers
```

- classifiers/mi/supportVector/MIPolyKernel.html (1.2)
- classifiers/mi/supportVector/MIRBFKernel.html (1.2)

2006-04-10 23:16 fracpete

```
updated Javadoc
```

- classifiers/functions/supportVector/NormalizedPolyKernel.html (1.5)
- classifiers/functions/supportVector/PolyKernel.html (1.5)
- classifiers/functions/supportVector/RBFKernel.html (1.6)

2006-04-10 23:05 fracpete

```
Kernels are now accessible via the GenericObjectEditor and OptionHandlers
```

- classifiers/functions/supportVector/CachedKernel.html (1.5)
- classifiers/functions/supportVector/Kernel.html (1.4)

2006-04-10 23:04 fracpete

```
added Kernels
```

- gui/GenericObjectEditor.html (1.52)

2006-04-10 23:03 fracpete

```
added Kernels
```

- gui/GenericPropertiesCreator.props (1.9)

2006-04-10 05:57 fracpete

```
added button for opening a separate dialog with the capabiliites (gets updated if properties sheet gets motified of a change, e.g., the base classifier of a meta classifier changed)
added serialVersionUIDs
```

- gui/PropertySheetPanel.html (1.16)

2006-04-05 01:12 fracpete

```
setClassifier now accepts the base classifier only if it can handle a numeric class (via Capability: NUMERIC_CLASS)
```

- classifiers/meta/RacedIncrementalLogitBoost.html (1.9)

2006-04-03 22:18 mhall

```
Fixed bug in tree output. Second number in the brackets at a leaf now reports the rms error as a percentage of the global standard deviation.
```

- classifiers/trees/m5/RuleNode.html (1.11)

2006-03-30 03:23 fracpete

```
added alt+left mousebutton as right click alternative for Mac users
```

- gui/SortedTableModel.html (1.3)

2006-03-30 03:22 fracpete

```
added alt+left mousebutton as right click alternative for Mac users
```

- gui/arffviewer/ArffPanel.html (1.5)

2006-03-29 06:36 fracpete

```
added serialVersionUIDs where necessary
added tags for Javadoc automation
fixed and updated Javadoc
```

- experiment/CSVResultListener.html (1.11)
- experiment/ClassifierSplitEvaluator.html (1.26)
- experiment/CostSensitiveClassifierSplitEvaluator.html (1.14)
- experiment/CrossValidationResultProducer.html (1.15)
- experiment/DatabaseResultProducer.html (1.16)
- experiment/Experiment.html (1.24)
- experiment/InstancesResultListener.html (1.9)
- experiment/LearningRateResultProducer.html (1.7)
- experiment/PairedTTester.html (1.33)
- experiment/RandomSplitResultProducer.html (1.18)
- experiment/RegressionSplitEvaluator.html (1.21)
- experiment/RemoteExperiment.html (1.14)

2006-03-29 06:36 fracpete

```
added serialVersionUIDs where necessary
added tags for Javadoc automation
fixed and updated Javadoc
```

- experiment/AveragingResultProducer.html (1.16)

2006-03-29 04:50 fracpete

```
added serialVersionUIDs where necessary
fixed and updated Javadoc
added support for RELATIONAL attribute type -> opens another editor for a particular cell value (which contains an Instances object)
```

- gui/arffviewer/ArffSortedTableModel.html (1.2)
- gui/arffviewer/ArffTableCellRenderer.html (1.3)
- gui/arffviewer/ArffTableModel.html (1.5)
- gui/arffviewer/ArffViewer.html (1.7)
- gui/arffviewer/ArffViewerMainPanel.html (1.3)
- gui/arffviewer/FileChooser.html (1.3)

2006-03-29 04:49 fracpete

```
cleaned up code (removed commented out and obsolete code)
added serialVersionUIDs where necessary
added tags for Javadoc automation
fixed and updated Javadoc
```

- core/KDTree.html (1.8)
- core/LinearNN.html (1.2)

2006-03-28 22:00 fracpete

```
added serialVersionUIDs where necessary
added tags for Javadoc automation
fixed and updated Javadoc
```

- core/converters/SerializedInstancesLoader.html (1.10)
- core/converters/SerializedInstancesSaver.html (1.4)

2006-03-28 21:50 fracpete

```
added serialVersionUIDs where necessary
added tags for Javadoc automation
fixed and updated Javadoc
```

- core/stemmers/NullStemmer.html (1.4)
- core/stemmers/Stemming.html (1.2)

2006-03-28 21:26 fracpete

```
added serialVersionUIDs where necessary
added tags for Javadoc automation
fixed and updated Javadoc
```

- core/converters/ArffLoader.html (1.11)
- core/converters/ArffSaver.html (1.4)
- core/converters/C45Loader.html (1.11)
- core/converters/C45Saver.html (1.4)
- core/converters/CSVLoader.html (1.10)
- core/converters/CSVSaver.html (1.4)
- core/converters/DatabaseConnection.html (1.3)

2006-03-28 05:04 fracpete

```
handles errors more gracefully now
```

- core/GlobalInfoJavadoc.html (1.4)
- core/OptionHandlerJavadoc.html (1.5)
- core/TechnicalInformationHandlerJavadoc.html (1.3)

2006-03-28 05:03 fracpete

```
added methods for object instantiation
```

- core/Javadoc.html (1.4)

2006-03-28 05:02 fracpete

```
added tags for Javadoc automation
fixed and updated Javadoc
```

- core/AllJavadoc.html (1.2)

2006-03-28 03:58 fracpete

```
added tags for Javadoc automation
fixed and updated Javadoc
```

- clusterers/CheckClusterer.html (1.3)

2006-03-28 03:57 fracpete

```
added tags for Javadoc automation
fixed and updated Javadoc
```

- classifiers/trees/lmt/LogisticBase.html (1.6)

2006-03-28 02:18 fracpete

```
added serialVersionUIDs where necessary
added tags for Javadoc automation
fixed and updated Javadoc
```

- classifiers/bayes/net/estimate/BMAEstimator.html (1.6)
- classifiers/bayes/net/estimate/BayesNetEstimator.html (1.2)
- classifiers/bayes/net/estimate/DiscreteEstimatorBayes.html (1.4)
- classifiers/bayes/net/estimate/DiscreteEstimatorFullBayes.html (1.2)
- classifiers/bayes/net/estimate/MultiNomialBMAEstimator.html (1.7)
- classifiers/bayes/net/estimate/SimpleEstimator.html (1.4)
- classifiers/bayes/net/search/SearchAlgorithm.html (1.6)
- classifiers/bayes/net/search/ci/CISearchAlgorithm.html (1.5)

2006-03-28 02:18 fracpete

```
added serialVersionUIDs where necessary
added tags for Javadoc automation
fixed and updated Javadoc
```

- classifiers/bayes/net/search/ci/ICSSearchAlgorithm.html (1.5)
- classifiers/bayes/net/search/fixed/FromFile.html (1.6)
- classifiers/bayes/net/search/fixed/NaiveBayes.html (1.4)
- classifiers/bayes/net/search/global/GeneticSearch.html (1.3)
- classifiers/bayes/net/search/global/GlobalScoreSearchAlgorithm.html (1.7)
- classifiers/bayes/net/search/global/HillClimber.html (1.7)
- classifiers/bayes/net/search/global/RepeatedHillClimber.html (1.4)
- classifiers/bayes/net/search/global/SimulatedAnnealing.html (1.3)
- classifiers/bayes/net/search/global/TabuSearch.html (1.3)
- classifiers/bayes/net/search/local/GeneticSearch.html (1.3)
- classifiers/bayes/net/search/local/HillClimber.html (1.7)
- classifiers/bayes/net/search/local/LAGDHillClimber.html (1.6)
- classifiers/bayes/net/search/local/RepeatedHillClimber.html (1.4)
- classifiers/bayes/net/search/local/Scoreable.html (1.2)
- classifiers/bayes/net/search/local/SimulatedAnnealing.html (1.3)
- classifiers/bayes/net/search/local/TabuSearch.html (1.3)

2006-03-27 03:13 fracpete

```
added serialVersionUID
fixed Javadoc
```

- core/Instance.html (1.23)

2006-03-24 05:08 eibe

```
Added changes from Marc Sumner's version written for PKDD paper (with some modifications).
```

- classifiers/trees/lmt/LMTNode.html (1.4)

2006-03-09 01:36 fracpete

```
integrated Lin's fixes
```

- classifiers/mi/SimpleMI.html (1.3)

2006-03-08 21:11 fracpete

```
fixed Javadoc
```

- datagenerators/classifiers/regression/Expression.html (1.3)

2006-03-08 21:05 fracpete

```
fixed Javadoc
```

- core/FindWithCapabilities.html (1.2)

2006-03-08 21:04 fracpete

```
fixed Javadoc
```

- experiment/InstanceQuery.html (1.18)

2006-03-08 02:28 fracpete

```
fixed Javadoc (for automation)
added serialVersionUID where necessary
```

- classifiers/trees/REPTree.html (1.23)

2006-03-08 02:24 fracpete

```
fixed Javadoc (for automation)
added getTechnicalInformation()
```

- classifiers/trees/J48.html (1.4)

2006-03-08 02:23 fracpete

```
fixed Javadoc (for automation)
```

- classifiers/trees/DecisionStump.html (1.20)

2006-03-08 02:22 fracpete

```
fixed Javadoc
added serialVersionUID
cleaned up imports
```

- classifiers/trees/j48/C45PruneableClassifierTree.html (1.13)

2006-03-08 02:22 fracpete

```
fixed Javadoc
added serialVersionUID
```

- classifiers/trees/j48/PruneableClassifierTree.html (1.10)

2006-03-08 02:21 fracpete

```
fixed Javadoc
added serialVersionUID
cleaned up imports
```

- classifiers/trees/j48/ClassifierTree.html (1.19)

2006-03-08 02:19 fracpete

```
fixed Javadoc (for automation)
added getTechnicalInformation()
```

- classifiers/trees/m5/M5Base.html (1.13)

2006-03-08 02:19 fracpete

```
fixed Javadoc
```

- classifiers/trees/m5/PreConstructedLinearModel.html (1.4)

2006-03-08 02:18 fracpete

```
fixed Javadoc (for automation)
```

- classifiers/rules/ZeroR.html (1.13)

2006-03-08 02:17 fracpete

```
fixed Javadoc
```

- classifiers/rules/Rule.html (1.4)

2006-03-08 02:17 fracpete

```
fixed Javadoc
added serialVersionUID
```

- classifiers/rules/RuleStats.html (1.5)

2006-03-08 02:14 fracpete

```
fixed Javadoc (for automation)
added getTechnicalInformation()
```

- classifiers/rules/NNge.html (1.5)

2006-03-08 02:12 fracpete

```
fixed Javadoc (for automation)
added serialVersionUID where necessary
```

- classifiers/rules/ConjunctiveRule.html (1.12)

2006-03-08 02:10 fracpete

```
fixed Javadoc (for automation)
added getTechnicalInformation()
added serialVersionUID where necessary
```

- classifiers/misc/HyperPipes.html (1.17)

2006-03-08 02:07 fracpete

```
fixed Javadoc (for automation)
added getTechnicalInformation()
```

- classifiers/mi/TLD.html (1.2)

2006-03-08 02:07 fracpete

```
fixed Javadoc (for automation)
added getTechnicalInformation()
```

- classifiers/mi/TLDSimple.html (1.2)

2006-03-08 02:06 fracpete

```
fixed Javadoc (for automation)
added getTechnicalInformation()
```

- classifiers/mi/MIWrapper.html (1.2)

2006-03-08 02:03 fracpete

```
fixed Javadoc (for automation)
```

- classifiers/mi/MILR.html (1.2)

2006-03-08 02:03 fracpete

```
fixed Javadoc (for automation)
added getTechnicalInformation()
```

- classifiers/mi/MINND.html (1.2)

2006-03-08 02:02 fracpete

```
fixed Javadoc (for automation)
added getTechnicalInformation()
```

- classifiers/mi/MIDD.html (1.2)

2006-03-08 02:01 fracpete

```
fixed Javadoc (for automation)
added getTechnicalInformation()
```

- classifiers/mi/MDD.html (1.2)

2006-03-08 02:00 fracpete

```
fixed Javadoc (for automation)
```

- classifiers/meta/Vote.html (1.9)

2006-03-08 01:59 fracpete

```
fixed Javadoc (for automation)
```

- classifiers/meta/ThresholdSelector.html (1.36)

2006-03-08 01:57 fracpete

```
fixed Javadoc (for automation)
```

- classifiers/meta/RegressionByDiscretization.html (1.32)

2006-03-08 01:56 fracpete

```
fixed Javadoc (for automation)
```

- classifiers/meta/RandomCommittee.html (1.9)

2006-03-08 01:54 fracpete

```
fixed Javadoc (for automation)
```

- classifiers/meta/MultiScheme.html (1.21)

2006-03-08 01:54 fracpete

```
fixed Javadoc (for automation)
cleaned up Imports
added serialVersionUIDs where necessary
```

- classifiers/meta/MultiClassClassifier.html (1.41)

2006-03-08 01:51 fracpete

```
fixed Javadoc (for automation)
```

- classifiers/meta/FilteredClassifier.html (1.24)

2006-03-08 01:49 fracpete

```
fixed Javadoc (for automation)
added getTechnicalInformation()
```

- classifiers/meta/CVParameterSelection.html (1.32)

2006-03-08 01:48 fracpete

```
fixed Javadoc (for automation)
```

- classifiers/meta/CostSensitiveClassifier.html (1.24)

2006-03-08 01:46 fracpete

```
fixed Javadoc (for automation)
```

- classifiers/meta/AttributeSelectedClassifier.html (1.22)

2006-03-08 01:32 fracpete

```
fixed Javadoc (for automation)
```

- classifiers/functions/SimpleLinearRegression.html (1.7)

2006-03-08 01:31 fracpete

```
fixed Javadoc (for automation)
```

- classifiers/functions/RBFNetwork.html (1.6)

2006-03-08 01:30 fracpete

```
fixed Javadoc (for automation)
```

- classifiers/functions/MultilayerPerceptron.html (1.4)

2006-03-08 01:29 fracpete

```
fixed Javadoc (for automation)
```

- classifiers/functions/LinearRegression.html (1.21)

2006-03-08 01:00 fracpete

```
fixed Javadoc (for automation)
added getTechnicalInformation()
cleaned up imports
```

- classifiers/bayes/net/BIFReader.html (1.10)

2006-03-08 00:38 fracpete

```
fixed Javadoc (for automation)
cleaned up Imports
```

- classifiers/bayes/net/BayesNetGenerator.html (1.10)

2006-03-08 00:36 fracpete

```
fixed Javadoc (for automation)
added getTechnicalInformation()
```

- classifiers/bayes/NaiveBayesSimple.html (1.17)

2006-03-08 00:29 fracpete

```
fixed Javadoc (for automation)
```

- classifiers/bayes/BayesNet.html (1.28)

2006-03-08 00:26 fracpete

```
added handling of "soft-capabilities", i.e., dependencies
```

- classifiers/MultipleClassifiersCombiner.html (1.4)
- classifiers/SingleClassifierEnhancer.html (1.5)

2006-03-08 00:18 fracpete

```
fixed Javadoc (for automation)
added serialVersionUIDs where necessary
added getTechnicalInformation() where applicable
```

- clusterers/EM.html (1.35)
- clusterers/MakeDensityBasedClusterer.html (1.8)
- clusterers/SimpleKMeans.html (1.25)

2006-03-08 00:16 fracpete

```
fixed Javadoc
```

- clusterers/DensityBasedClusterer.html (1.5)

2006-03-08 00:09 fracpete

```
fixed Javadoc
added serialVersionUID
```

- gui/sql/ConnectionPanel.html (1.2)

2006-03-06 22:48 fracpete

```
Gabi's fixes
```

- core/AlgVector.html (1.5)

2006-03-05 23:01 fracpete

```
now implements weka.core.CapabilitiesHandler
```

- associations/Associator.html (1.6)

2006-03-03 03:27 fracpete

```
automated Javadoc generation (globalInfo, options)
added TechnicalInformation-handling where possible
```

- datagenerators/ClassificationGenerator.html (1.2)
- datagenerators/ClusterDefinition.html (1.2)
- datagenerators/ClusterGenerator.html (1.4)
- datagenerators/DataGenerator.html (1.3)
- datagenerators/RegressionGenerator.html (1.2)
- datagenerators/Test.html (1.3)
- datagenerators/classifiers/classification/BayesNet.html (1.2)
- datagenerators/classifiers/classification/LED24.html (1.3)
- datagenerators/classifiers/classification/RDG1.html (1.2)
- datagenerators/classifiers/classification/RandomRBF.html (1.2)
- datagenerators/classifiers/regression/MexicanHat.html (1.2)

2006-03-03 03:27 fracpete

```
automated Javadoc generation (globalInfo, options)
added TechnicalInformation-handling where possible
```

- datagenerators/clusterers/SubspaceCluster.html (1.2)
- datagenerators/clusterers/SubspaceClusterDefinition.html (1.2)

2006-03-03 01:31 fracpete

```
automated Javadoc generation (globalInfo, options)
added TechnicalInformation-handling where possible
```

- attributeSelection/BestFirst.html (1.26)
- attributeSelection/CfsSubsetEval.html (1.22)
- attributeSelection/ChiSquaredAttributeEval.html (1.10)
- attributeSelection/ClassifierSubsetEval.html (1.14)
- attributeSelection/ExhaustiveSearch.html (1.12)
- attributeSelection/GainRatioAttributeEval.html (1.18)
- attributeSelection/GeneticSearch.html (1.15)
- attributeSelection/GreedyStepwise.html (1.7)
- attributeSelection/InfoGainAttributeEval.html (1.16)
- attributeSelection/OneRAttributeEval.html (1.16)

2006-03-03 01:31 fracpete

```
automated Javadoc generation (globalInfo, options)
added TechnicalInformation-handling where possible
```

- attributeSelection/Ranker.html (1.23)
- attributeSelection/SymmetricalUncertAttributeEval.html (1.17)

2006-03-03 01:28 fracpete

```
fixed javadoc
cleaned up code
```

- gui/beans/xml/XMLBeans.html (1.6)

2006-02-23 22:46 fracpete

```
adapted to work with "Open" button
```

- gui/visualize/ThresholdVisualizePanel.html (1.3)

2006-02-23 19:51 fracpete

```
removed CRs from source code (was mixture of CRLF and LF)
```

- classifiers/bayes/net/VaryNode.html (1.3)

2006-02-23 04:48 fracpete

```
used for paper references (can also generate BibTeX output)
```

- core/TechnicalInformationHandler.html (1.1)

2006-02-21 02:17 fracpete

```
added JCheckBox for debug flag in DatabaseUtils
```

- gui/DatabaseConnectionDialog.html (1.5)

2006-02-21 02:17 fracpete

```
added support for debug flag from DB-Connection dialog
```

- gui/experiment/ResultsPanel.html (1.40)
- gui/experiment/SimpleSetupPanel.html (1.11)

2006-02-20 21:57 fracpete

```
fixed bug with interfaces (return null as superclass)
```

- core/ClassDiscovery.html (1.3)

2006-02-20 01:33 mhall

```
Changes from Jimmy
```

- gui/boundaryvisualizer/BoundaryPanel.html (1.20)

2006-02-19 23:27 fracpete

```
added get-methods for m_NewBatch and m_FirstBatchDone
```

- filters/SimpleFilter.html (1.2)

2006-02-19 23:17 fracpete

```
added customized getCapabilities method
updated buildClassifier method
cleaned up imports
```

- classifiers/trees/j48/NBTreeClassifierTree.html (1.2)

2006-02-19 22:16 fracpete

```
implements now CapabilitiesHandler
```

- classifiers/rules/part/MakeDecList.html (1.14)

2006-02-19 21:00 fracpete

```
now implements the weka.core.CapabilitiesHandler interface
returns a Capabilties object that only says that it can handle datasets without a class, other capabilities have to be enabled by derived classes
```

- clusterers/Clusterer.html (1.11)

2006-02-19 20:56 fracpete

```
implements now the weka.core.CapabilitiesHandler interface
returns by default a Capabilities object that has NO capabilities. derived classifiers need to enable the necessary capabilities (so far every classifier could handle anything by default, which made it hard to add new features)
```

- classifiers/Classifier.html (1.13)

2006-02-19 20:38 fracpete

```
interfaces to return Capabilities objects
```

- core/CapabilitiesHandler.html (1.1)
- core/MultiInstanceCapabilitiesHandler.html (1.1)

2006-02-19 20:34 fracpete

```
extended JList that contains JCheckBox items
```

- gui/CheckBoxList.html (1.1)

2006-02-14 22:44 fracpete

```
branches:  1.1.2;
added GUI editor for SimpleDateFormat class
```

- gui/SimpleDateFormatEditor.html (1.1)

2006-02-02 03:39 fracpete

```
Ashraf's bug fix to stop KDTree using the class attribute (if set) in its tree
structure.
```

- core/EuclideanDistance.html (1.7)

2006-01-19 01:51 fracpete

```
added new constructor with the readable text of the Tag instead of the index
means easier parsing of symbolic options from commandline
```

- core/SelectedTag.html (1.8)

2006-01-07 23:43 fracpete

```
visualization plugin interface for the Explorer/Classify panel
by Jeffery Grajkowski
```

- gui/visualize/plugins/VisualizePlugin.html (1.1)

2005-12-22 04:22 fracpete

```
added up/down button
```

- gui/experiment/DatasetListPanel.html (1.18)

2005-12-22 04:21 fracpete

```
added up/down button
```

- gui/experiment/AlgorithmListPanel.html (1.15)

2005-12-22 04:19 fracpete

```
added up/down button
```

- gui/GenericArrayEditor.html (1.16)

2005-12-22 04:18 fracpete

```
helper class for swing component JList
```

- gui/JListHelper.html (1.1)

2005-12-22 04:08 fracpete

```
no real use, besides educational
```

- filters/NullFilter.html (1.8)
